# Optimization of a Simplified and Effective Analytical Method of Pesticide Residues in Mealworms (*Tenebrio molitor* Larvae) Combined with GC–MS/MS and LC–MS/MS

**DOI:** 10.3390/molecules25153518

**Published:** 2020-07-31

**Authors:** Leesun Kim, Sujin Baek, Kyungae Son, Eunsun Kim, Hyun Ho Noh, Danbi Kim, Min-seok Oh, Byeong-chul Moon, Jin-Ho Ro

**Affiliations:** 1 Chemical Safety Division, National Institute of Agricultural Sciences, RDA, Iseo-myeon, Wanju-gun, Jeollabuk-do 55365, Korea; twosuns@korea.kr (L.K.); bsj920@gmail.com (S.B.); noh1983@korea.kr (H.H.N.); danbi6334@korea.kr (D.K.); minseok84@korea.kr (M.-s.O.); moonbc@korea.kr (B.-c.M.); 2 Agromaterial Assessment Division, National Institute of Agricultural Sciences, RDA, Iseo-myeon, Wanju-gun, Jeollabuk-do 55365, Korea; sky199@korea.kr; 3 Industrial Insect Division, National Institute of Agricultural Sciences, RDA, Iseo-myeon, Wanju-gun, Jeollabuk-do 55365, Korea; eunsunny88@korea.kr

**Keywords:** mealworms, pesticide residue, QuEChERS, GC–MS/MS, LC–MS/MS

## Abstract

An effective analytical method was optimized for residues including chlorpyrifos–methyl, deltamethrin, fenoxanil, thiobencarb and fludioxonil in mealworms, the larval form of *Tenebrio molitor*. They are listed for pest control during wheat cultivation and can be found in wheat-bran feed for growing mealworms in South Korea. Analytes were extracted using acetonitrile and salt packet. Four clean-up methods ((1) MgSO_4_ + 25 mg PSA + 25 mg C18; (2) MgSO_4_ + 50 mg PSA + 50 mg C18; (3) EMR-lipid^TM^ tube; and (4) 10 mL *n*-hexane) were investigated and the method (1) was selected due to its robustness. Low-temperature precipitation of fat and proteins improved the recoveries. Recoveries from the Method (1) were satisfying with 70–120% with <20% relative SD at a spiking level of 0.01 mg/kg. With the simultaneous sample preparation, fenoxanil, thiobencarb and fludioxonil were analyzed by liquid chromatography tandem mass spectrometry (LC–MS/MS) and chlorpyrifos–methyl and deltamethrin by gas chromatography tandem mass spectrometry (GC–MS/MS). Quantification limits for LC–MS/MS and GC–MS/MS were 0.5 and 2.5 μg/L, respectively. No pesticides of interest were detected in 30 real samples collected across the nation. However, the data can be provided for establishing maximum residue limits for the pesticides in mealworms in response to the positive list system.

## 1. Introduction

As the global population increases, more enhanced and efficient food production is required [1]. For example, in disadvantaged social parts or during natural disasters or social conflict times, protein and other nutritional deficiencies are more prevalent. In addition, the production of beef and pork requires large amount of energy and causes various environmental issues such as groundwater contamination [2]. In this regard, edible insects including beetles, cockroaches, grasshoppers, honeybees, mealworms, silkworms, termites and wasps have drawn attention as alternative protein food sources because insects have great nutritional values (fat, 14.4–33.7%; proteins, 39.3–64.4%) [3] and can offer an environmentally friendly and efficient opportunity to cope with food insecurity among vulnerable people [4]. However, in developed countries, insects can be perceived as an unpleasant food or feed source and people are often unwilling to use them as such. Therefore, the food safety of edible insects can play an important role in altering the negative perception of advanced nations about insects [5]. In addition, some European nations such as the Netherlands and Belgium now pay more attention to the promotion and production of insect-derived food, which are now sold on their local markets [6].

**Figure 1:**
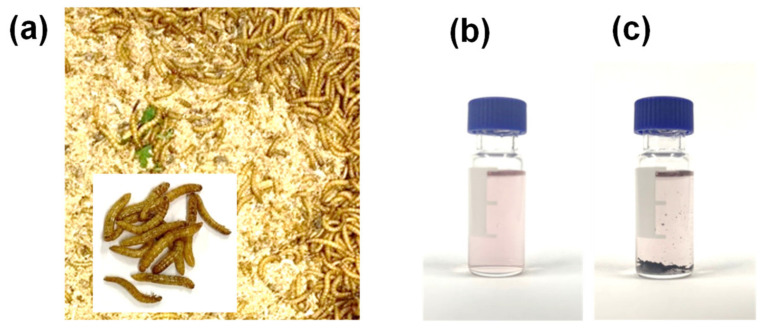
(**a**) Mealworms grown in wheat bran; (**b**) acetonitrile (MeCN) extract of mealworms and (**c**) MeCN extract of mealworms after 24 h in freezer (−20 °C).

The Korean Beneficial Insects Laboratory carries out studies about animal feed, beneficial insects and natural enemies [7] because the use of edible insects as a viable food or feed source can provide many economic and environmental benefits. Many Korean farmers harvest various edible insects, including grasshoppers, crickets, mealworms, silkworms, rhino beetle larvae and white-spotted chafer larvae which the Korean Ministry of Food and Drug Safety allowed to use for human use [7]. Several insect-derived products, such as chocolate and protein powders or bars are already produced in South Korea. The “Safe Rearing Manual for Edible Insects” published by the National Institute of Agricultural Sciences (2017) specifies the feed for rearing edible insects to monitor food contamination. Previous studies reported that there are various possibilities for the pollutants in feed to be transferred into insects [6]. For example, mealworms are recommended to be fed with wheat bran (Figure 1a). Pesticides used for the harvest and storage of wheat can also be transferred into mealworms. In addition, maximum residue limits (MRLs) in edible insects including mealworms are not yet established in South Korea because edible insects are newly developed ingredients for feed and food as protein sources. To our best knowledge, few studies optimized the analytical methods of organic pollutants, including pesticides, in edible insects. Therefore, analytical methods of pesticides in edible insects and setting up MRLs are urgently required for consumer safety.

Out of five pesticides (chlorpyrifos–methyl, deltamethrin, fenoxanil, thiobencarb and fludioxonil) selected in this study, a mixture of chlorpyrifos–methyl and deltamethrin or fenoxanil is usually used on stored wheat and for the structural treatment of grain storages [8]. In case of chlorpyrifos–methyl, the European Commission withdrew its authorization for plant protection products because of its possible genotoxicity and developing neurotoxicity by February 2020 [9]. Thiobencarb and fludioxonil are generally recommended for wheat cultivation by Korean National Institute of Agricultural Sciences (2019). The pesticide analysis in dried mealworms can be challenging due to high contents of fat (35.42%) and proteins (52.82%) in the matrix [3]. In particular, chromatographic methods for the determination of pesticide residue concentration generally require thorough lipid elimination before the sample is introduced into the system [10]. Therefore, the clean-up procedure should be optimized to effectively remove sample-derived matrices.

Quick, easy, cheap, effective, rugged and safe (QuEChERS) methods were modified for the extraction of multiresidues from various sample matrices since their introduction [11,12,13], combined with gas chromatography tandem mass spectrometry (GC–MS/MS) and liquid chromatography tandem mass spectrometry (LC–MS/MS). Many studies reported that various adsorbents were adopted to remove high-fat-content matrices, such as in avocados [14,15], cereals [16], honey bees [17], peanut oil [18], soybean-based products [19] and vegetable oils [20,21] (Table 1). However, some issues derived from high fat content in very complex matrices still remains because each pesticide can show different behavior in different matrices [21]. Though primary secondary amine (PSA) and magnesium sulfate (MgSO_4_) are basic adsorbents for the removal of strong acids and polyacidic compounds mainly derived from aqueous samples, C18, Z-sep, Z-sep plus and enhanced matrix removal (EMR)-lipid^TM^ were investigated for the effective removal of matrix-derived lipids [20,22]. Previous studies improved the recoveries of lipophilic compounds by changing the ratio of solvent/sample to reduce matrix effects derived from a wide range of food matrices [20]. In the extraction step, *n*-hexane was also used to remove fatty matrices from the sample extract [23]. In particular, sorbent compositions or ratio in the clean-up procedure can be more critical to eliminate fatty residues from complex matrices [21]. These studies also showed that the ratio of adsorbents can change clean-up efficiency because target compounds can be retained by sorbents.

This study was designed to optimize an effective and simplified method for the simultaneous analysis of chlorpyrifos–methyl, deltamethrin, fenoxanil, thiobencarb and fludioxonil in mealworms using GC–MS/MS and LC–MS/MS. The QuEChERS extraction and dispersive solid phase extraction (d-SPE) clean-up procedures were investigated for more effective and practical analysis to achieve reasonable recoveries. In terms of accuracy, precision and limit of quantitation (LOQ), the optimized method was validated. In the end, the method was applied for residue analysis of real samples provided by 30 different mealworm farms in South Korea.

## 2. Results and Discussion

### 2.1. Evaluation of Sample-Extraction Solvent

**Figure 2:**
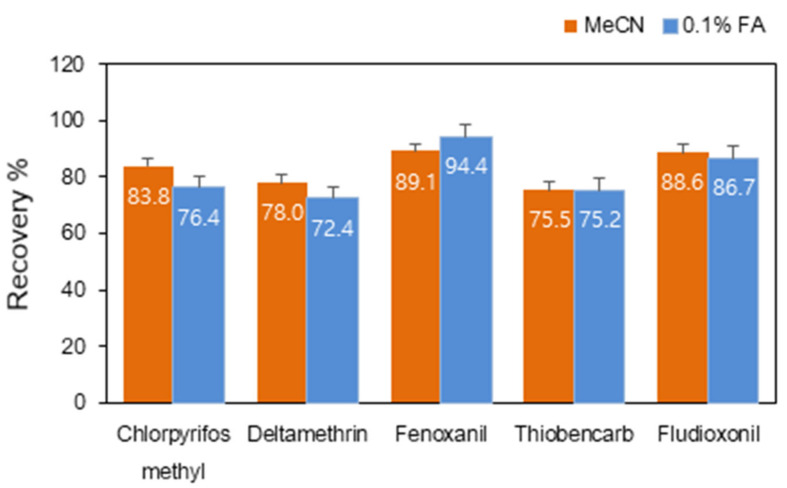
Recoveries of each pesticide obtained from two different extraction solvent, acetonitrile (MeCN) and 0.1% formic acid (CH_2_O_2_; FA) MeCN.

Extraction solvents were investigated to select the proper solvent to simultaneously extract chlorpyrifos–methyl, deltamethrin, fenoxanil, thiobencarb and fludioxonil. The addition of formic acid (CH_2_O_2_; 0.1%) to acetonitrile (MeCN) and MeCN alone were compared. Clean-up method (1) was used for this experiment. Figure 2 shows recoveries of the target compounds obtained from both solvent methods at the fortification level (0.01 mg/kg). Recoveries were ranged between 70% and 120% with relative SD (RSD) ≤ 20% in both extraction procedures. Since the introduction of QuEChERS [11], a wide range of studies also reported the modified methods [13,20]. Acetate (AOAC 2007.1)- and citrate (EN15662)-buffered QuEChERS methods are generally used for the extraction of hundreds of pesticides. In order to extract pH-sensitive compounds, formic or acetic acid is often added to the extraction solvent in extraction procedure [26,27]. Acid is known to assist in stabilizing degradable pesticides under basic conditions [26,28].

On the basis of a statistical test (ANOVA), the recoveries of each pesticide using MeCN were not differently from those using 0.1% CH_2_O_2_ (*p* > 0.05) except for chlorpyrifos–methyl. On the basis of these data, it was deduced that citrate buffered QuEChERS method is enough to control the pH of the solvent for the extraction of the target analytes in this study. Therefore, MeCN without CH_2_O_2_ was chosen as an extraction solvent, considering that an analytical method must be rapid in terms of sample throughput and turnaround time, cost-effective, ready-to-perform and rugged, as well as attaining high quality results with a wide range of analytical scope. Previous studies showed that chlorpyrifos–methyl, deltamethrin, fenoxanil, thiobencarb and fludioxonil were successfully extracted from samples with high fat contents using MeCN alone [10,20]. It was also demonstrated that the recoveries of each pesticide using MeCN (93.6%) are not different from those using 0.1% CH_2_O_2_ (93.9%) to extract over 300 pesticides from soybeans, brown rice and other vegetables [10].

### 2.2. Optimization of Clean-Up Procedures

One of the greatest difficulties in extracting pesticide residues from complex matrices with high fat contents such as oil, fish and milk, is the interference of co-extracts in the chromatographic response. To solve this issue, most published studies investigated several clean-up steps, which are involved with solid phase and/or liquid–liquid extraction and low temperature precipitation (Table 2).

**Figure 3:**
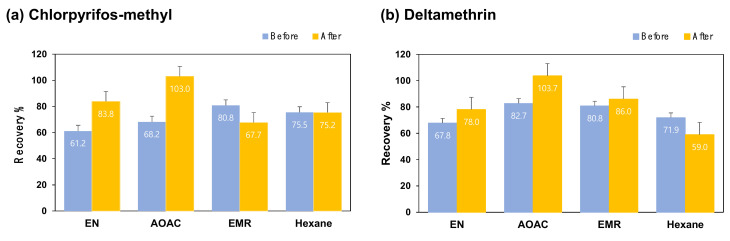
Recoveries of (**a**) chlorpyrifos–methyl and (**b**) deltamethrin obtained from four different clean-up methods before and after cooling extracts at −20 °C for 24 h. EN—150 mg MgSO_4_ + 25 mg PSA + 25 mg C18; AOAC—150 mg MgSO_4_ + 50 mg PSA + 50 mg C18; EMR-lipid^TM^ and 10 mL *n*-hexane.

To remove fat and proteins from the extract with high fat contents, low temperature precipitation was used in the clean-up procedure for pesticide analysis [24,29]. In this experiment, the clean-up procedure of the extract was performed as soon as the sample was extracted and 24 h after the samples were left at −20 °C. Each extract is shown in Figure 1b,c. On the bottom of the extract shown in Figure 1c, the blackish residues were observed. They may have been derived from insect cuticles containing proteins. The recoveries of chlorpyrifos–methyl and deltamethrin obtained from both clean-up procedures are shown in Figure 3a,b. Recoveries obtained from the EN and AOAC clean-up procedures after the matrix was precipitated at −20 °C were increased by 10 to 20%. The proposed clean-up method showed that the recoveries were increased from 61.2 to 83.8% for chlorpyrifos–methyl and from 67.8 to 78.0% for deltamethrin (ANOVA, *p* < 0.05), which demonstrated that low temperature precipitation is required to effectively remove lipids from the insect derived samples. One of the previous studies reported that the low-temperature-precipitation method allowed for removing 90.6% fats from the palm-oil extract [24].

**Figure 4:**
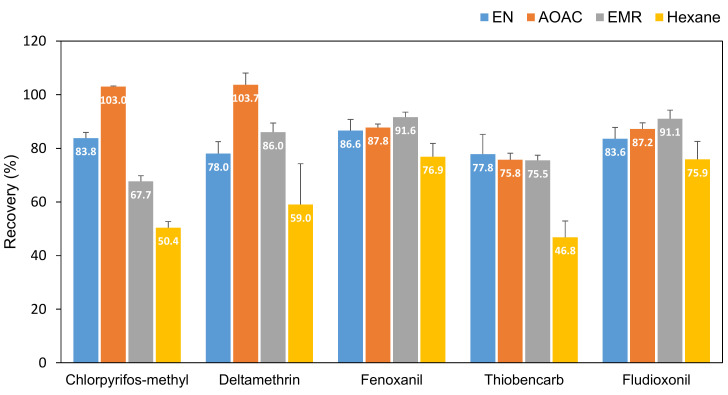
Recoveries of each pesticide obtained from four different clean-up methods. (1) EN—150 mg MgSO_4_ + 25 mg primary secondary amine (PSA) + 25 mg C18; (2) acetate (AOAC)—150 mg MgSO_4_ + 50 mg PSA + 50 mg C18; (3) EMR-lipid^TM^ and (4) 10 mL *n-*hexane.

Figure 4 shows differences in the recoveries of the pesticide obtained during optimization of different types of clean-up procedures and low-temperature precipitates in this study. As a reversed phase sorbent, C18 is known to retain lipids, minerals and vitamins. In this study, the EN mixture (150 mg MgSO_4_ + 25 mg PSA + 25 mg C18) was selected as an optimized clean-up method, which is consistent with most of studies that used C18 to remove lipids from the samples (Table 2). The mixture achieved a simplified and robust analysis of the pesticides of interest. In particular, compared with the AOAC mixture (150 mg MgSO_4_ + 50 mg PSA + 50 mg C18), large differences were not observed. Recoveries of chlorpyrifos–methyl and deltamethrin were increased by 22.9% and 32.9% by adding extra 25 mg PSA and 25 mg C18. The previous study also selected the AOAC mixture for the clean-up procedure for deltamethrin [30]. Deltamethrin is a difficult compound because the sensitivity is low on the chromatographic space. When it is extracted from a fatty matrix (such as in olive oil), a high solvent ratio gave a good recovery rate of deltamethrin [31]. However, recoveries of all the compounds obtained by the first mixture still satisfied SANTE/2017/11813 (70–120%) [30] and the statistical test showed that there were no significant differences between the two results (ANOVA, *p* > 0.05). A reduced amount of the clean-up absorbent was preferred due to it being an environmentally friendly and low-cost procedure. Previous study reported that recoveries of deltamethrin (78.1–82.4% ± 2.2–3.5%), chlorpyrifos–methyl (96.3–97.6% ± 1.4–5.8%), fenoxanil (95.6–97.0% ± 2.3–3.1%), fludioxonil (102.9–103.6% ± 3.2–17.9%) and thiobencarb (99.1–99.5% ± 1.9–6.4%) in soybeans were successfully achieved using d-SPE containing PSA and MgSO_4_ [10]. Considering that mealworms have much higher fat (35.42%) and protein (52.82%) contents compared with soybeans (fat 20% and protein 40%), extra adsorbents such as C18 should be required.

The enhanced matrix removal-lipid (EMR-lipid^TM^) method was investigated in this experiment because a previous study reported that EMR-lipid^TM^ gave the best recoveries for 255 pesticides [20]. EMR-lipid^TM^ is a newly developed sorbent as an alternative of Z-Sep but its composition was not disclosed by the producer. In this study, low recoveries of some target compounds were observed compared with the AOAC mixture. Recoveries of chlorpyrifos–methyl and thiobencarb were lowered by 19.2% and 3.0% respectively, while recoveries of deltamethrin, fenoxanil and fludioxonil were increased by 10%, 5.8% and 8.9%. It was reported that EMR-lipid^TM^ eliminated lipids from matrices (e.g., olive oil and beans) with substantial analyte loss [21]. A previous study reported that regarding the separation of triphenylene and chrysene, no differences were observed been the mixture of 150 mg MgSO_4_ + 25 mg PSA + 25 mg C18, Z-sep and EMR-lipid^TM^. The EN mixture was selected because of the higher response of the target pesticides and the availability of low-cost and easy-to-use kits [22].

In this study, *n*-hexane was also tested to remove excessive fat contents in the extracts without further clean-up procedure because it has greater capability for extracting oil [32]. It aggressively mixes with the oil and washes it out without disturbing fiber, protein, sugar and undesired gums. However, in this study, recoveries of all analytes of interest were much lower by the addition of *n*-hexane compared with other clean-up methods. The recoveries of chlorpyrifos–methyl (39.8%), deltamethrin (24.4%), fenoxanil (11.2%), thiobencarb (39.8%) and fludioxonil (9.2%) was lowered by adding *n*-hexane.

The addition of *n*-hexane successfully removed interferences such as wax [23]. However, recoveries for alachlor and dimethoate (4%) and for carbofuran and coumaphos (12%) among 19 analytes were lowered by the addition of *n*-hexane. Diazinon gave a greater difference (22%). For other analytes, better recoveries were obtained by applying a mixture of deionized water (DW)/MeCN/*n*-hexane in the extraction procedure, compared with the mixture of DW and MeCN.

### 2.3. Matrix Effect, Method Validation and Real Sample Analysis

The matrix effect (ME) was determined for each target pesticide residue in mealworms. The mean value of the matrix effect for each pesticide was less than 0.1%. This could probably be achieved due to the use of matrix-matched calibration curves when quantitative analysis was performed. Significant interferences from the sample matrix that could lead to peak identification or quantification issues were not observed.

The proposed QuEChERS process in this study was evaluated for the target pesticides from mealworms. Recovery studies were carried out at two spiking levels of 0.01 and 0.1 mg/kg for GC–MS/MS and 0.005 and 0.01 mg/kg for LC–MS/MS at replicates (n = 3). Calibration curves presented linearity in the range of 1–50 μg/kg for GC–MS/MS and 0.5–25 μg/kg for LC–MS/MS. All calibration curves displayed linearity with r^2^ > 0.99. All studied pesticides studied gave recoveries between 70% and 120% with RSD lower than 20% for both fortification levels, which satisfied with the SANTE guidelines (Table 3). The method limit of quantification (MLOQs) for GC–MS/MS and LC–MS/MS was 2.5 and 0.5 μg/L, respectively. The optimized method was applied to the real samples collected from 30 farms across the nation and no target pesticides were detected (Table S4). However, the optimized method gave several benefits such as the following: (a) minimized lipid residues in the final extract through MeCN extraction/salting-out; (b) reduced sample preparation time by omitting the evaporation step; (c) minimized matrix effect by injecting diluted sample to both GC–MS/MS and LC–MS/MS; (d) eliminated lipid residues through low-temperature precipitation and quick clean-up process for instrumental analysis; and (e) maintained system integrity and decreased instrumental downtime by using column backflush program in the GC system.

## 3. Materials and Methods

### 3.1. Chemicals and Consumables

Pesticide standards, namely, chlorpyrifos–methyl, deltamethrin, fenoxanil, fludioxonil and thiobencarb were purchased from Dr. Ehrenstofer (LGC, Ltd., Teddington, UK). All analyte standards had purity of ≥98%. MeCN, *n*-hexane, DW and concentrated CH_2_O_2_ were of analytical reagent grade and purchased from Thermo Fisher Scientific (Waltham, MA, USA). Individual stock solutions of each standard were diluted in MeCN at a concentration of 1000 µg/mL. The QuEChERS packet containing 4 g MgSO_4_, 1 g NaCl, 1 g sodium citrate and 0.5 g disodium citrate sesquihydrate, EMR-lipid^TM^, d-SPE containing 150 mg of MgSO_4_, 25 mg of PSA and 25 mg of C18 (EN15662) and 150 mg of MgSO_4_, 50 mg of PSA and 50 mg of C18 (AOAC) and homogenizer were obtained from Agilent Technologies (Santa Clara, CA, USA).

### 3.2. Sample Treatment

Organic mealworms, *Tenebrio molitor* larvae, were provided by Industrial Insect Division, National Institute of Agricultural Sciences in South Korea. Samples were pulverized after addition of dry ice using a blender and stored in a freezer at −20 °C until analysis. The sample (5 g) was added into a 50 mL of polypropylene centrifuge tube. Each sample was left for 30 min after it was moisturized with 10 mL of DW. MeCN (10 mL) or MeCN with 0.1% CH_2_O_2_ (10 mL) was added to the sample with a ceramic homogenizer before it was dynamically agitated by a Mini G Genogrinder (SPEX. CentiPrep^®^ 1500) for 1 min. After the QuEChERS packet was added to the sample, it was left in ice to control the heat for 15–20 min and then shaken using a Mini G for 1 min before centrifugation at 3500 rpm for 15 min. The prepared samples were stored in the freezer (−20 °C) for 24 h to physically precipitate lipids and protein residues.

For clean-up evaluation, three different clean-up sorbent compositions were tested and *n*-hexane (10 mL) was used without further clean-up. Details of the clean-up materials are listed in Table 1. The extract was transferred into a d-SPE centrifuge tube and dynamically shaken before centrifugation (12,000 rpm) at 4 °C for 15 min. In the case of EMR-lipid^TM^, 5 mL of extracts was added to the water- activated tube (15-mL tube) for clean-up for centrifugation. For both methods, the sample was filtrated through a 0.22-µm syringe filter (GHP Acrodisc 13 mm, Pall life sciences, New York, NY, USA) before preparation of matrix-matched standards and samples for instrumental analysis. For *n*-hexane clean-up, the supernatant was transferred into a new tube (50 mL) with 10 mL of *n*-hexane. The mixture was vigorously agitated using mini Geno grinder (CentiPrep 1500, SPEX. Metuchen, NJ, USA) for 1 min before centrifugation (3500 rpm) at 4 °C. The bottom layer (1 mL) was filtered through a syringe filter for instrument analysis.

### 3.3. Instrumental Analysis

Samples were simultaneously analyzed using and GC (Agilent Technologies 7890B) combined with MS/MS (Agilent technologies 700 °C GC-TQ) and LC (Exion LC, SCIEX Framingham, MA, USA) combined with MS/MS (TQ5500, SCIEX Framingham, MA, USA). The GERSTEL MultiPurpose Sampler (PTV inlet, cooled injection system CIS, GERSTEL, Linthicum, MD, USA) which allows for large-volume injections, was installed in the GC system.

GC chromatographic separation of chlorpyrifos–methyl and deltamethrin was carried out with a nonpolar stationary phase column (HP-5MS, 15 m × 0.25 mm i.d. × 0.25 µm *df*; Agilent Technologies). A guard column (HP-5MS, 5 m × 0.25 mm i.d. × 0.25 µm *df*; Agilent Technologies) was connected to the inlet using a union to backflush residues that remained on the column to prevent cross- contamination between each sample run. Conditions for GC analysis were as follows: injection temperature was at 60 °C, the injection volume was 4.0 µL and splitless, which was a large-volume injection by CIS. Initial oven temperature was set at 60 °C (for 1.5 min), raised at a rate of 50 °C/min up to 150 °C, increased to 240 °C at 8 °C/min (for 3 min) and lastly raised to 280 °C at 50 °C/min (for 6 min). The carrier gas was ultra-high-purity helium (99.999%) at a constant flow of 1.2 mL/min. Solvent delay was 3.0 min for the optimized method. The ion-source and interface temperatures and electron impact ionization energy were adjusted at 280 °C, 280 °C and 70 eV, respectively. The mass spectrometer was run in a multiple reaction monitoring for quantitative analysis. Retention times and characteristic mass fragments of GC-amenable compounds for qualitative and quantitative analysis are in Table S1 in the Supplementary Materials. Full-scan MS data were obtained in the range of *m*/*z* 50–550 in order to acquire the fragmentation spectra of each compound.

The LC chromatographic separation of fenoxanil, thiobencarb and fludioxonil was carried out with a reverse phase column (Halo C18, 2.7 µm, 2.1 mm i.d. × 100 mm, USA). Flow rate was 0.1 mL/min, injection volume was 1 µL and ion spray voltage +5500 V. Pressures of nebulizer, heater and curtain gas were 50, 50 and 25 psi, respectively. Mobile Phase A was 0.1% CH_2_O_2_ in DW and Mobile Phase B was 0.1% CH_2_O_2_ in methanol. The gradient program is shown in Table S2. Retention times and characteristic mass fragments for LC-amenable compounds are shown in Table S3.

### 3.4. Analytical Method Validation and Real Sample Analysis

The optimized method in this study was validated on the basis of SANTE/11,813/2017 criteria [30]. Its accuracy and precision were assessed through mean recoveries (%) and relative standard deviation (RSD, %), respectively, at two spiking levels of 0.01 and 0.1 mg/kg for GC analysis and 0.005 and 0.01 mg/kg for LC analysis. Matrix-matched standards were employed to reduce instrument signal enhancement or suppression (matrix effect) by the addition of blank sample extracts to each standard solution. For quantitative analysis, the matrix-matched standards were prepared at the levels of 2.5, 5, 10, 25 and 50 ng/g for GC–MS/MS and at the levels of 1, 2.5, 5, 10 and 25 ng/g for LC–MS/MS. Matrix-dependent limit of quantitation (LOQ) and linearity were assessed by each calibration curve of matrix-matched standards. The lowest concentration having an S/N ratio of a quantifier ion peak above 10 was determined as LOQ.

Matrix effect (ME) (%) had to be determined because it is one of the main issues in the MS/MS analysis. In this study, it was obtained as average percent enhancement or suppression by dividing the slope of the matrix-matched calibration curves by that of the standard calibration curve in MeCN. Equation (1) was applied,

(1) $$\mathrm{ME}\left(\%\right)=\left(\frac{slope\;of\;matrix-matched\;standards\;curve}{slope\;of\;solvent\;standards\;curve}-1\right)\times 100$$

A value of 100% indicates no matrix effect. If it was less than 100%, this indicated matrix suppression. If it was more than 100%, it indicated matrix enhancement. Signal enhancement was generally observed in the GC–MS/MS determination of previous studies [10,33]. Therefore, optimized ME is an important aspect that has an effect on quantitative accuracy in multiresidue analysis. The ME is generally derived by interaction between active sites of the column or liner and target compounds or matrices in the GC analysis. The nature of target compounds and samples, analytical instruments and extraction process also influences the degree of the ME. In particular, when the target compound is co-eluted with matrix components in the MS/MS analysis, interaction between the target and matrices in the ionization step can cause decreasing or increase the ion amount.

Real sample analysis was carried out using the analytical method optimized in this study. Thirty different farmers across South Korea kindly donated their mealworms in June 2019. For statistical analysis, Excel (Microsoft 2013) was used for one-way ANOVA tests.

## 4. Conclusions

A simple and practical method for the determination of pesticide residues in mealworms by GC–MS/MS and LC–MS/MS was described in this study. The method was optimized and validated to effectively monitor pesticides in mealworms that are already available in the Korean market. The simplicity and applicability of the optimized method allow its use for routine analysis of chlorpyrifos–methyl, deltamethrin, thiobencarb and fludioxonil in edible-insect matrices, with enough sensitivity to quantify low concentrations below limits established by the positive list system (spiking level 0.01 mg/kg). It has the advantages of being simple, inexpensive and rapid, with time-effective and environmentally friendly analysis. Even though no target pesticides were detected in the 30 different farm samples across the nation, the developed method can be applied for continuously monitoring pollutants in any edible insects and insect derived feed or food.

## Figures and Tables

**Table 1:** List of studies which used various clean-up sorbents to remove lipids from the samples with high fat contents.

Samples/ Weight	Analytes	QuEChERS^1^ Packet/Solvent	Clean-Up (Sample Amount)	Instrument	Reference
Peanut oil/5 g	9 OP^2^	0.50 g Na_2_SO_4_/10 mL MeCN^3^	100 mg MWCNTs^10^/1 g neutral alumina	GC–MS^16^	[18]
Palm oil/3 g	7 pesticides	Original^4^ 7 g DW^5^ + 10 mL MeCN	d-SPE^11^ (15 mL) 750 mg MgSO_4_ + 250 mg PSA^12^ + 250 mg C18 + 250 mg GCB^13^	LC–TOF–MS^17^	[24]
Honeybee/1 g	19 pesticides	Original 10 mL DW + 10 mL MeCN + 3 mL *n*-hexane	d-SPE (1 mL) 150 mg MgSO_4_ + 25 mg PSA	LC–MS/MS^18^	[23]
Cereals/5 g	200 pesticides	(1) AOAC^6^ / (2) EN^7^ 10 mL DW + 15 mL MeCN (1% AA^8^)	d-SPE (8 mL/6 mL) (1) 1200 mg MgSO_4_ + 400 mg PSA + 400 mg C18 (2) 900 mg MgSO_4_ + 150 mg PSA + 150 mg C18	GC–MS/MS^19^	[16]
Olive oil/0.5 g	138 pesticides	AOAC 5 mL DW + 30 mL MeCN (1% AA)	d-SPE (2 mL) 150 mg MgSO_4_ + 50 mg PSA + 50 mg C18	GC–MS/MSLC–MS/MS	[25]
Edible vegetable oils/0.5, 1, 2, 5 g	255 pesticides	4 g NaCl 5 mL DW + 10 mL MeCN	d-SPE (6.5 mL) (1) 150 mg PSA + 150 mg C18 (2) 250 mg PSA + 250 mg C18 (3) 250 mg PSA + 250 mg C18 + 15 mg GCB (4) 250 mg PSA + 250 mg C18 + 50 mg GCB (5) EMR-lipid^14^	GC–MS/MS	[20]
Salmon, shrimps, mussels, cutlet, bacon/5 g	4 PAHs^9^	EN 5 mL DW + 10 mL MeCN	d-SPE (6.5 mL) (1) 900 mg MgSO_4_ + 150 mg PSA + 150 mg C18, (2) Z-Sep^15^ (3) EMR-lipid	GC–MS/MS	[22]

^1^ QuEChERS—quick, easy, cheap, effective, rugged and safe; ^2^ OP—organophosphorus pesticides ^3^ MeCN—acetonitrile; ^4^ original—4 g of MgSO_4_ and 1 g of NaCl; ^5^ DW—deionized water; ^6^ AOAC—6 g MgSO_4_, 1.5 g sodium acetate; ^7^ EN—4 g MgSO_4_, 1 g NaCl, 1 g sodium citrate and 0.5 g disodium citrate sesquihydrate; ^8^ AA—acetic acid; ^9^ PAHs—polycyclic aromatic hydrocarbons; ^10^ MWCNTs—multi-walled carbon nanotubes; ^11^ d-SPE—dispersive solid phase extraction; ^12^ PSA—primary secondary amine; ^13^ GCB—graphitized carbon black; ^14^ EMR-lipid—enhanced matrix removal-lipid; ^15^ Z-Sep—zirconia; ^16^ GC–MS—gas chromatography mass spectrometry; ^17^ LC–TOF–MS—liquid chromatography time of flight mass spectrometry; ^18^ LC–MS/MS—liquid chromatography tandem mass spectrometry; ^19^ GC–MS/MS—gas chromatography tandem mass spectrometry.

**Table 2:** Recoveries of each pesticide at two spiking levels.

Target Pesticide	Spiking Level (mg/kg)	Mean (%)	RSD^1^ (%)
Chlorpyrifos-methyl	0.01	82.5	3.0
	0.1	114.8	1.6
Deltamethrin	0.01	93.8	6.5
	0.1	84.9	5.7
Fenoxanil	0.005	87.6	4.6
	0.01	112.9	4.2
Thiobencarb	0.005	87.6	4.6
	0.01	83.3	1.3
Fludioxonil	0.005	82.0	7.2
	0.01	76.9	3.4

^1^ RSD: relative standard deviation.

**Table 3:** List of clean-up procedure investigated in this study.

No	Sorbent Compositions	Sample Volume	Product Name
1	150 mg MgSO_4_, 25 mg PSA^1^, 25 mg C18	1 mL	EN
2	150 mg MgSO_4_, 50 mg PSA, 50 mg C18	1 mL	AOAC
3	^2^	5 mL	EMR-lipid^TM.3^
4	10 mL *n*-hexane	10 mL	

^1^ PSA—primary secondary amine; ^2^ the contents were not disclosed by the producer; ^3^ EMR-lipid^TM^—enhanced matrix removal-lipid method.

## Supplementary Materials

The following are available online, Table S1: The optimized GC-MS/MS parameters including retention time (t_R_, min) of each pesticide, selected reaction monitoring transitions and collision energy (CE, V), Table S2: The gradient program of liquid chromatography. Table S3: The optimized LC-MS/MS parameters including retention time (t_R_, min) of each pesticide, selected reaction monitoring transitions and collision energy (CE, V), DP, EP and CXP. Table S4: Concentrations of five pesticides analyzed in the real mealworm samples collected from 30 different farms in South Korea.
