# Supplementary material for: Optimization of a Simplified and Effective Analytical Method of Pesticide Residues in Mealworms (Tenebrio molitor Larvae) Combined with GC–MS/MS and LC–MS/MS
Source: Molecules. 2020 Jul 31;25(15):3518. doi: 10.3390/molecules25153518 (PMC7435900; doi:10.3390/molecules25153518)
Supplement: Supplementary file 1 [file molecules-25-03518-s001.pdf]

Supplementary Materials for

Optimization of a simplified and effective analytical method of pesticide residues in mealworms (*Tenebrio moliter* Larvae), combined with GC-MS/MS and LC-MS/MS

Leesun Kim[1], Sujin Baek[1], Kyungae Son[2], Eunsun Kim[3], Hyun Ho Noh[1], Danbi Kim[1], Min-seok Oh[1], Byeong-chul Moon[1], Jin-Ho Ro[1,*]

[1]*Chemical Safety Division, National Institute of Agricultural Sciences, RDA, Iseo-myeon, Wanju-gun, Jeollabuk-do 55365, South Korea*
[2]*Agromaterial Assessment Division, National Institute of Agricultural Sciences, RDA, Iseo-myeon, Wanju-gun, Jeollabuk-do 55365, South Korea*
[3]*Industrial Insect Division, National Institute of Agricultural Sciences, RDA, Iseo-myeon, Wanju-gun, Jeollabuk-do 55365, South Korea*

Content **Page**

Table S1	The optimized GC-MS/MS parameters including retention time (t_R, min) of each pesticide, selected reaction monitoring transitions, and collision energy (CE, V).	S2
Table S2	The gradient program of liquid chromatography.	S3
Table S3	The optimized LC-MS/MS parameters including retention time (t_R, min) of each pesticide, selected reaction monitoring transitions, and collision energy (CE, V), DP, EP, and CXP.	S3
Table S4	Concentrations of five pesticides analyzed in the real mealworm samples collected from 30 different farms in South Korea.	S4

[*]Corresponding author. Tel: +82 63 238 2318; fax: +82 63 238 3837.
E-mail address: jhro@korea.kr

Table S1. The optimized GC-MS/MS parameters including retention time (t_R, min) of each pesticide, selected reaction monitoring transitions, and collision energy (CE, V).

**Compounds**	**t_R (min)**	**Precursor ion>Product ion (CE, V)**	
		Quantifier	Qualifier
chlropyrifos-methyl	8.8	285.8>271.0 (16)	287.8>93.0 (26)
deltamethrin	18.1	180.9>152 (26)	252.8>93 (20)

Table S2. The gradient program of liquid chromatography.

Time (min)	Initial	11	15	16	20
A (%)	75	10	10	75	75
B (%)	25	90	90	25	25

Table S3. The optimized LC-MS/MS parameters including retention time (t_R, min) of each pesticide, selected reaction monitoring transitions, and collision energy (CE, V), DP, EP, and CXP.

Compounds	t_R (min)	Quantifier / Qualifier	DP	EP	CE	CXP
fenoxanil	13.65	329.084> 302.100	91	10	25	4
		329.084>86.100	69	10	67	4
thiobencarb	14.70	258.100>125.000	76	10	45	4
		258.100>89.000	81	10	49	4
fludioxonil	11.74	246.895>180.000	115	10	42	15
		246.895>126.000	115	10	42	11

Table S4. Residue concentrations of five pesticides analyzed in the real mealworm samples collected from 30 different farms in South Korea.

No	Farm	Concentration of pesticide residue (mg/kg)				
		Chlorpyrifos-methyl	Deltamethrin	Fenoxanil	Thiobencarb	Fludioxonil
1	YW1-1	< 0.01	< 0.01	< 0.005	< 0.005	< 0.005
2	YW2-1	< 0.01	< 0.01	< 0.005	< 0.005	< 0.005
3	YJ1-1	< 0.01	< 0.01	< 0.005	< 0.005	< 0.005
4	YI1-1	< 0.01	< 0.01	< 0.005	< 0.005	< 0.005
5	SC1-1	< 0.01	< 0.01	< 0.005	< 0.005	< 0.005
6	HC1-1	< 0.01	< 0.01	< 0.005	< 0.005	< 0.005
7	GJ1-1	< 0.01	< 0.01	< 0.005	< 0.005	< 0.005
8	GJ2-1	< 0.01	< 0.01	< 0.005	< 0.005	< 0.005
9	GJ3-1	< 0.01	< 0.01	< 0.005	< 0.005	< 0.005
10	SJ1-1	< 0.01	< 0.01	< 0.005	< 0.005	< 0.005
11	GYC1-1	< 0.01	< 0.01	< 0.005	< 0.005	< 0.005
12	GYC2-1	< 0.01	< 0.01	< 0.005	< 0.005	< 0.005
13	PH1-1	< 0.01	< 0.01	< 0.005	< 0.005	< 0.005
14	PH2-1	< 0.01	< 0.01	< 0.005	< 0.005	< 0.005
15	DJ1-1	< 0.01	< 0.01	< 0.005	< 0.005	< 0.005
16	US1-1	< 0.01	< 0.01	< 0.005	< 0.005	< 0.005
17	GS1-1	< 0.01	< 0.01	< 0.005	< 0.005	< 0.005
18	DY1-1	< 0.01	< 0.01	< 0.005	< 0.005	< 0.005
19	DY2-1	< 0.01	< 0.01	< 0.005	< 0.005	< 0.005
20	DY3-1	< 0.01	< 0.01	< 0.005	< 0.005	< 0.005
21	JS101	< 0.01	< 0.01	< 0.005	< 0.005	< 0.005
22	JS2-1	< 0.01	< 0.01	< 0.005	< 0.005	< 0.005
23	GC1-1	< 0.01	< 0.01	< 0.005	< 0.005	< 0.005
24	GC2-1	< 0.01	< 0.01	< 0.005	< 0.005	< 0.005
25	GC3-1	< 0.01	< 0.01	< 0.005	< 0.005	< 0.005
26	CYC1-1	< 0.01	< 0.01	< 0.005	< 0.005	< 0.005
27	CYC2-1	< 0.01	< 0.01	< 0.005	< 0.005	< 0.005
28	CYC3-1	< 0.01	< 0.01	< 0.005	< 0.005	< 0.005
29	CYC4-1	< 0.01	< 0.01	< 0.005	< 0.005	< 0.005
30	CYC5-1	< 0.01	< 0.01	< 0.005	< 0.005	< 0.005
